# Demographic consequences of reproductive interference in multi-species communities

**DOI:** 10.1186/s12898-018-0201-0

**Published:** 2018-11-06

**Authors:** Janice J. Ting, Asher D. Cutter

**Affiliations:** Department of Ecology & Evolutionary Biology, University of Toronto, Toronto, ON M5S3B2 Canada

**Keywords:** Caenorhabditis, Reproductive interference, Interference competition, Sperm

## Abstract

**Background:**

Reproductive interference can mediate interference competition between species through sexual interactions that reduce the fitness of one species by another. Theory shows that the positive frequency-dependent effects of such costly errors in mate recognition can dictate species coexistence or exclusion even with countervailing resource competition differences between species. While usually framed in terms of pre-mating or post-zygotic costs, reproductive interference manifests between individual *Caenorhabditis* nematodes from negative interspecies gametic interactions: sperm cells from interspecies matings can migrate ectopically to induce female sterility and premature death. The potential for reproductive interference to exert population level effects on *Caenorhabditis* trait evolution and community structure, however, remains unknown.

**Results:**

Here we test whether a species that is superior in individual-level reproductive interference (*C. nigoni*) can exact negative demographic effects on competitor species that are superior in resource competition (*C. briggsae* and *C. elegans*). We observe coexistence over six generations and find evidence of demographic reproductive interference even under conditions unfavorable to its influence. *C. briggsae* and *C. elegans* show distinct patterns of reproductive interference in competitive interactions with *C. nigoni*.

**Conclusions:**

These results affirm that individual level negative effects of reproductive interference mediated by gamete interactions can ramify to population demography, with the potential to influence patterns of species coexistence separately from the effects of direct resource competition.

**Electronic supplementary material:**

The online version of this article (10.1186/s12898-018-0201-0) contains supplementary material, which is available to authorized users.

## Background

Resource competition, predation, and mutualism/parasitism span the gamut of traditional views about ecologically-important interspecies interactions [e.g. 1–5]. Interspecies sexual interactions like misdirected courtship, heterospecific mating, and hybridization, however, may not just be ‘noise’ [6]. Instead, reproductive interference (RI) can result from reduced fitness in one or both species through direct harm, wasted time or energy, or forfeited gametes from interspecies sexual interactions as a form of interference competition [7–9]. Similar to other interspecies interactions, RI can influence community structure and evolutionary trajectories from species displacement versus coexistence, specialization (e.g. spatial, temporal, or habitat), and reproductive character displacement [7, 10, 11]. Evolutionary responses of species to harmful sexual interactions between them thus can represent an interspecies byproduct of intraspecies sexually antagonistic evolution [12]. Even if interspecies sexual interactions affect individual fitness (‘component RI’), however, theory shows that community characteristics are not guaranteed to be perturbed (‘demographic RI’) [10]; if demographic RI occurs, however, then some mechanism of component RI must also be present [10]. Thus, it is crucial to determine the incidence of both component and demographic RI to understand the potential for harmful interspecies sexual interactions to influence population dynamics and community composition.

To predict whether or not species can coexist with one another, it is important to consider both reproductive interference and resource competition. In competitive dynamics, the defining characteristic of RI is its positive frequency dependence: the rarer species will suffer disproportionately from costly interspecies sexual interactions [13]. As a result, species coexistence becomes less likely even with weak effects of RI [13–15]. The outcome of this frequency dependent effect of RI is simplest to conceive when the costs of RI are symmetric between species, rather than one species suffering disproportionately. Whether species can coexist with one another, however, depends especially strongly on the degree of the asymmetry of RI compared to the asymmetry of resource competition [16], and RI is typically observed in empirical studies to be asymmetric, where one species is more adversely affected than the other [7]. As a result, a species that is superior in resource competition could coexist with or even be excluded by another species that is superior in reproductive interference [16]. This dynamic is especially pertinent to modern-day communities because, for example, conditions favourable for biological species invasions due to global climate change may set the stage for both resource competition and reproductive interference to contribute to the likelihood of species invasion success [7, 17, 18]. Human impacted environments also may aggravate the incidence of interspecies sexual encounters by disrupting pre-mating cues (e.g. noise and light pollution on courtship [19, 20]).

Reproductive interference often is studied from the perspective of hybrid progeny production between species, though mating and pre-mating modes of RI also have been characterized (signal jamming, heterospecific rivalry, misdirected courtship, heterospecific mating attempts, erroneous female choice) [7, 21, 22]. Incompatibilities between species at the gametic level also are known to make important contributions to the speciation process [23]. Consequently, reproductive interference also could manifest at the gametic level, during the post-mating pre-zygotic phase, contributing to component RI, or demographic RI, or both. If females of a species commonly suffer harm from having mated with males of other species, then we would expect selection to favor the evolution of tolerance or resistance to the harm, or, of pre-mating barriers to preclude exposure to the negative effects, as for reproductive character displacement [9, 24]. Thus reproductive interference has the potential to drive within-species mate choice dynamics as an evolutionary outcome of between-species interactions.

Failure of species recognition from weak mate preference/avoidance in *Caenorhabditis* nematodes leads different species to mate with one another and transfer sperm [25–28]. These interactions can exact harm on female individuals, inducing sterility and reduced longevity [28], thus demonstrating component RI. The primary source of this individual-level reproductive interference is heterospecific male sperm that displace existing conspecific sperm and that migrate to ectopic regions of the gonad or even into the body cavity [28]. This form of gametic reproductive interference does not affect all species equally (asymmetrical reproductive interference) and has no known effect on males (sex-biased) [28]. Moreover, *Caenorhabditis* mating, even with conspecific males, can be costly in the form of reduced longevity and lifetime reproductive output [29, 30], for example, with mating-induced physical damage to the cuticle from repetitive spicule insertions by males that potentially increase vulnerability to bacterial or fungal infections [31, 32]. Most species of *Caenorhabditis* are dioecious (male/female), with three known exceptions, including *C. elegans* and *C. briggsae* used in this study, in which populations are androdioecious (males and self-fertile hermaphrodites; hermaphrodites incapable of inseminating other hermaphrodites) [33]. In addition, experimental populations of *C. elegans* and *C. briggsae* can be genetically manipulated to be either purely hermaphroditic or fully dioecious [34]. Purely hermaphroditic populations typically have much higher growth rates than mixed-sex populations [35], potentially leading to displacement or extinction of outcrossing species through simple competition for resources. However, such species would represent disproportionate targets of sperm-induced harm by virtue of the female morphology and sensitivity to heterospecific harm of hermaphrodite individuals [28], such that demographic RI could disadvantage them despite their superior resource competition [18, 36]. In principle, such interactions could contribute to patterns of local and regional species richness in these organisms that have highly patchy resource distributions [33, 37] and potentially drive the evolution of mate choice traits. Here we test for multi-generation demographic effects of asymmetric gametic RI in *Caenorhabditis* nematodes by quantifying community composition and population growth rates in experimental species assemblages.

## Methods

### Overview

We created experimental communities comprised of one or two species of *Caenorhabditis* nematodes and quantified their abundances after ~ 6 generations (15 days); we use the term ‘community’ broadly to define groups of organisms of different species or of the same species that differ in a fundamental reproductive phenotype that share common resources and habitat. We distinguished species with the aid of integrated transgenic fluorescent markers expressed in pharyngeal muscle (green fluorescent protein, GFP, in *C. elegans* and *C. briggsae*; *Discosoma* sp. red fluorescent protein, DsRed, in *C. nigoni*; Table 1). By rearing the animals with ad libitum food availability, we minimized resource competition with the aim of testing for interference competition from the sensitivity of population growth rate estimates to the presence of other species [16]. Specifically, interspecies matings of *C. nigoni* males to *C. elegans* and *C. briggsae* lead to ectopic migration of sperm that induces sterility and premature death of inseminated individuals [28], termed ‘component reproductive interference (RI),’ which we hypothesized could depress population growth rates of *C. elegans* and *C. briggsae* in mixed-species communities (‘demographic RI’). Females of *C. nigoni* do not suffer detectable component RI effects [28]. All three species also exhibit strong post-zygotic reproductive isolation with one another [38–41]. By using isogenic and isofemale strains for each species, we limit the communities to ecological dynamics and exclude potential coevolutionary responses.

**Table 1:** Species and strain genotypes used to create experimental communities

Species	Strain: genotype^a^	Phenotype	Tendency to receive male sperm
*C. briggsae*	JU610: mfIs5[*Cbr*-*egl*-*17*::GFP; Cel-*myo*-*2*::GFP]	GFP-marked ‘wild type’ facultative outcrossing hermaphrodites	Moderate
*C. briggsae*	VX0262: *Cbr*-*she*-*1(v83)*	‘Feminized’ females and males	High
*C. briggsae*	DY199: *Cbr*-*lin*-*39(bh23)*	‘Vulvaless’ selfing hermaphrodites	Low
*C. nigoni* ^b^	VX0090: mfIs42[Cel-*sid*-*2*; Cel-*myo*-*2*::DsRed2]	DsRed-marked females and ‘harmful’ males	High
*C. elegans*	PD4790: mIs12 II [*myo*-*2*p::GFP + *pes*-*10*p::GFP + F22B7.9p::GFP]	GFP-marked ‘wild type’ facultative outcrossing hermaphrodites	Moderate
*C. elegans*	JK574: *fog*-*2(q71)*	‘Feminized’ females and males	High
*C. elegans*	PS436: *let*-*60(sy93)*	‘Vulvaless’ selfing hermaphrodites	Low

^a^*Cbr*-*she*-*1* [44], *Cbr*-*lin*-*39* [80], PD4790 [81], *fog*-*2* [82], *let*-*60* [55]

^b^Same isofemale strain genotype of *C. nigoni* was used in both experiments as the source of heterospecific male sperm

### Experimental community composition

We constructed seven community types for each of two experimental blocks that involved either *C. elegans* or *C. briggsae*. Two of the treatments types contained just a single isogenic strain of a single species (Table 1): wildtype *C. elegans* (PD4790, GFP transgene marker in N2 genetic background) or wildtype *C. briggsae* (JU610, GFP transgene marker in AF16 genetic background) and *C. nigoni* (VX0090, DsRed transgene marker in JU1325 genetic background; repeated in both experimental blocks). *C. elegans* and *C. briggsae* populations are normally composed entirely of self-fertilizing hermaphrodites whereas *C. nigoni* populations are dioecious with an equal sex ratio of males to females.

The remaining five treatment types contained two-strain communities, such as wildtype *C. elegans* hermaphrodites mixed with *C. nigoni* (Table 1). Gonads of hermaphrodites first produce sperm in both *C. elegans* and *C. briggsae*, then switch to irreversible production of oocytes which enables self-fertilization [42]. Hermaphrodites are incapable of inseminating other hermaphrodites, and their behavior leads to poor mating efficacy even with conspecific males (< 1% male individuals) [27]. In communities containing these hermaphrodites mixed with heterospecific *C. nigoni*, therefore, we expect only a fraction of hermaphrodites to mate and receive harmful *C. nigoni* male sperm.

To experimentally increase the potential exposure of *C. elegans* and *C. briggsae* to harmful heterospecific male sperm, we constructed mixed communities containing ‘feminized’ hermaphrodites with an allele that transforms hermaphrodites into ‘females’ (Table 1). The *C. elegans* isogenic strain JK574 thus is dioecious with a 1:1 male:female sex ratio (*fog*-*2* knockout mutation in N2 genetic background) [35, 43]. Consequently, the females must mate to reproduce which may increase their risk of erroneous heterospecific mating and reproductive interference (similar feminizing effects for *C. briggsae* strain VX0262 with disruption of *Cbr*-*she*-*1* in an AF16 genetic background) [44]. To complement the interspecies community type, we also constructed communities comprised of wildtype and feminized strains of the same species for both *C. elegans* and *C. briggsae* (e.g. PD4790 with JK574; Table 1).

With the aim of creating an experimental treatment that minimizes the potential for sperm-mediated reproductive interference, we constructed communities of *C. nigoni* mixed with hermaphrodites of *C. elegans* or *C. briggsae* that are incapable of mating (Table 1). Specifically, we used isogenic strains of hermaphrodites that lacked a vulva and so can only produce self-progeny because they cannot be inseminated (*C. elegans* PS436 with genetic disruption of *let*-*60* in the N2 genetic background; *C. briggsae* DY199 with genetic disruption of *Cbr*-*lin*-*39* in the AF16 genetic background; Table 1). A consequence of the vulvaless phenotype is that eggs will hatch inside the hermaphrodite, leading to lower fitness and longevity than wildtype hermaphrodites [45]. To complement the interspecies community, we also constructed communities comprised of wildtype and vulvaless strains of the same species for both *C. elegans* and *C. briggsae* (Table 1).

### Founding, maintenance and assessment of ecological communities

We age-synchronized founding individuals at the first larval (L1) stage from each strain using a standard bleaching protocol [46], then estimated L1 density for each strain from three aliquots of 5 μL. By adjusting the concentration appropriately, we transferred 20 μL drops of L1s to found each community type with ~ 50 or ~ 100 individuals of a given strain (Table 2). We varied the starting densities among community types to match the number of reproducing hermaphrodites or females for each community, assuming a 1:1 sex ratio among L1 animals for dioecious strains. We maintained populations following modified standard *C. elegans* procedures [47], rearing at 25 °C on *E. coli* (OP50) food on 9 cm diameter NGM-lite agar Petri dishes with agar concentration increased to 2.2% to discourage animals from burrowing [46]. To ensure communities would have an over-abundance of food for a week, we pelleted concentrated overnight 1L B-broth cultures of *E. coli* by centrifugation of 50 mL aliquots at 5000 rpm for 10 min. The concentrated *E. coli* was re-suspended in 500 μL and spread onto the surface of 9 cm diameter agar plates, allowed to dry, and then grown overnight at 37 °C. After adding a founding community to an individual agar dish with food, Petri dishes were then sealed with Parafilm^®^ and placed in plastic boxes with moist Kimwipes^®^ to maintain humidity at 90–100%. We initiated 10 replicates per treatment (with two exceptions, one treatment with n = 9 and one with n = 12).

**Table 2:** Initial experimental composition of each community type

Phenotypes of *C. elegans* or *C. briggsae*^a^	Frequency of wildtype (%)	Frequency of *C. nigoni* (%)^b^	Number of individuals in founding community^c^
Wildtype hermaphrodites	100	0	50
None	0	100	100
Wildtype hermaphrodites	33	67	150
Vulvaless hermaphrodites	0	67	150
Feminized females and males	0	50	200
Wildtype and vulvaless hermaphrodites	50	0	100
Wildtype hermaphrodites and feminized females and males	33	0	150

^a^Each of the seven community types were initiated for blocks with *C. elegans* or with *C. briggsae*

^b^*C. nigoni* were excluded from three community types that contained only wildtype hermaphrodites or mixtures of conspecific genotypes with contrasting reproductive modes

^c^Initial population sizes were set to equalize the number of egg-laying individuals (females or hermaphrodites) to 50 for each species within each community type assuming 1:1 sex ratio for genotypes with males and females

We conducted just a single transfer of communities to fresh food, after 7 days (~ 3 generations), to minimize disruption to communities. None of the replicates had depleted their food supply or gone extinct prior to transfer and all replicates retained all strains that they started with, distinguishable by fluorescent markers. We then estimated animal abundance to calculate intrinsic growth rates for single-genotype communities according to an exponential growth model (see calculations below): after washing worms off each plate with 5 mL M9 buffer, we estimated density (worms/μL) from a 2 μL aliquot. Simultaneously, we transferred 20 μL from each replicate to a new 9 cm agar plate with food as described above (aliquot of 0.4%).

We terminated the experiment 15 days after inception (~ 6 generations; no extinctions), estimating densities and the proportional composition of distinct strains within each replicate community. As previously, we estimated density (worms/μL) from the average of three aliquots of 2 μL from a 5 mL wash of each dish with M9 buffer. We then concentrated the worms (1800 rcf for 2 min) and pipetted a 2 μL aliquot and 2 μL of 500 mM of sodium azide (to induce paralysis) onto a microscope slide with a 10% agarose pad, covered by a glass coverslip. We captured digital images of worms with white light and with red and green fluorescent channels at 4× or 10× magnification (depending on spatial distribution of worms) with a fluorescence compound microscope and camera (Olympus BX51 with DP80 camera, using Olympus cellSens Standard 1.14). We then manually marked the location of worms of each fluorescent phenotype on each image using Adobe Photoshop CS6 (minimum 100 worms per replicate). We counted the number of worms of each type by processing the marked images using ImageJ’s particle analysis function. In 3 of the 102 replicates of two-strain communities, one of the strains was not detected, despite both types being visible in qualitative scans of each replicate sample. Consequently, we used a pseudo-count of 1 for the rarer type in subsequent calculations for those three replicates.

We noticed that not all worms showed detectable expression of the DsRed marker in those communities comprised solely of *C. nigoni* that should show 100% of red individuals: 87.1% ± 1SE = 2.13 (experimental block with *C. briggsae*, *n *= 9), 80.3% ± 2.10 (experimental block with *C. elegans*, *n *= 10). By contrast, nearly all individuals expressed GFP (97.9% ± 6.38 in *C. briggsae*, *n *= 10; 99.8% ± 0.0013 in *C. elegans*, *n *= 10). Consequently, we numerically adjusted the relative abundance accordingly for *C. nigoni* in mixed communities to account for this imperfect detection, using a correction factor separately for the experimental blocks conducted with *C. elegans* and with *C. briggsae*.

### Growth rate estimation in ecological communities

We estimated the “community rate of growth” of the population mixtures for each replicate by solving for *r* in a simple continuous time model for exponential population growth, which assumes unlimited resources, using estimates of initial and final abundances. Specifically, given the starting number of worms in a treatment (*C*_0_) and the number after *t* generations (*C*_*t*_):

1 $$C_{t} = C_{0} e^{rt}$$

Solving for the intrinsic rate of growth (*r*) yields:

2 $$r = \frac{{\ln \left( {\frac{{C_{t} }}{{C_{0} }}} \right)}}{t}$$

We used the input number of founding individuals for the value of *C*_0_ and the number of worms estimated at the transfer point for *C*_*t*_, where we assume *t* = 3 generations. An ANOVA was then performed to test for differences in growth rate (*r*) among treatments. For single-strain communities, *r* represents the intrinsic rate of population growth for that strain; for multi-strain communities, *r* represents a metric of biomass accumulation as a combined measure of community-wide population growth across strains or species.

We could calculate *r* directly for single-strain treatments only for *C. nigoni* and for wildtype *C. elegans* and *C. briggsae*. To estimate growth rate (*r′*) for individual strains in mixed-strain communities that had genetically-transformed reproductive modes (obligatorily dioecious, obligately selfing), we used information about the relative abundances of each strain at the inception and end of the experiment in a model of exponential growth. Specifically, we related the relative abundances given frequency *P*_0_ of the competitor strain when the community was founded and the final frequency *P*_*t*_ of the competitor strain at the end of the experiment as

3 $$\frac{{P_{t} }}{{1 - P_{t} }} = \frac{{P_{0} e^{r t} }}{{(1 - P_{0} ) e^{{r^{\prime}t}} }}$$

(frequencies 1 − *P*_0_ and 1 − *P*_*t*_ for the focal strain; *t* = 6 generations). We then computed the estimated growth rate for the focal strain by solving for *r′* to yield:

4 $$r' = \left.\left[ {r t + \ln \left( {\frac{{P_{0} (1 - P_{t} ) }}{{(1 - P_{0} ) P_{t} }}} \right)} \right]\right/t$$

Using this approach, we estimated the growth rate of individual strains in each replicate for treatments of mixed-strain communities that contained obligate selfing (vulvaless) or obligate dioecious (feminized hermaphrodites) reproductive modes.

### Statistical analyses for ecological experiments

**Fig. 1:**
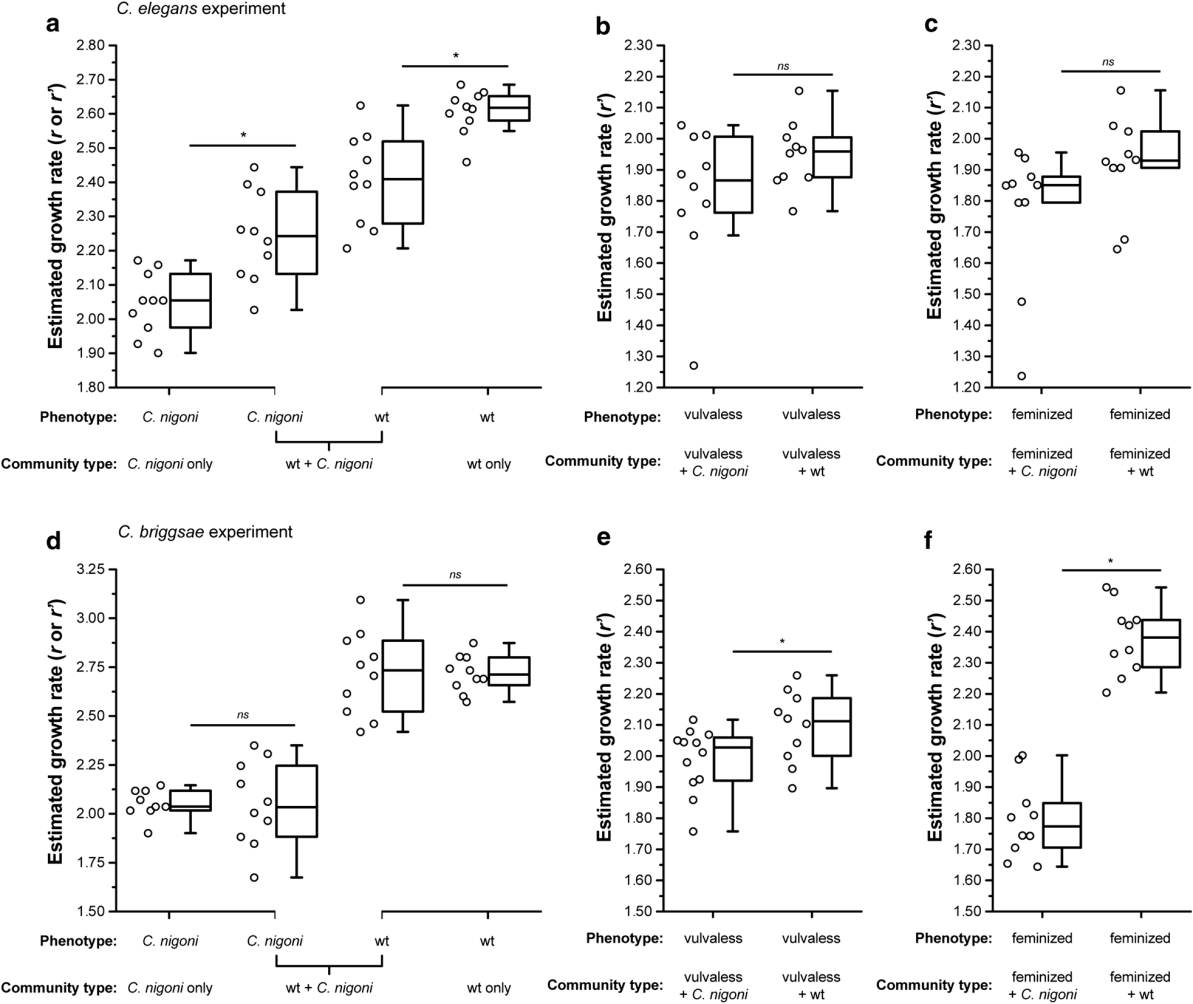
Presence of *C. nigoni* interferes with reproduction of *C. elegans* and *C. briggsae*. **a**–**c** Growth rate estimates for the *C. elegans* experimental block show significant differences in wildtype *C. elegans* (wt) growth rate when grown alone or in the presence of *C. nigoni*, but no effect of *C. nigoni* on growth rate estimates for vulvaless and feminized strains of *C. elegans*. **d**–**f** In the *C. briggsae* experimental block, growth rate estimates for both vulvaless and feminized strains of *C. briggsae* are reduced in the presence of *C. nigoni*, whereas wildtype *C. briggsae* growth rates were not significantly affected. Growth rate estimates for each species or reproductive phenotype in mixed communities (*r′*) used Eq. 4 that incorporates direct growth rate estimates (*r*) from pure strains based on Eq. 2. The growth rates from the different community types were compared using a *t* test with Bonferroni correction for multiple tests in (**a** wt comparison *t *= 4.22, df = 18, P ≤ 0.001; *C. nigoni* comparison *t *= − 3.86, df = 18, P ≤ 0.001) and (**d** wt comparison *t *= − 0.03, df = 12.25, P = 0.98; *C. nigoni* comparison *t *= 0.03, df = 11.25, P = 0.98) (α = 0.025), where asterisks (*) indicate differences (α = 0.05) and *ns* indicates non-significant differences (**b** *t *= − 1.59, df = 18, P = 0.13; **c** *t *= − 1.76, df = 18, P = 0.095; **e** *t *= − 2.22, df = 20, P = 0.038; **f** *t *= − 10.91, df = 18, P ≤ 0.001). Boxplot whiskers indicate 1.5*(interquartile range) and open circles show estimates for each replicate population

Statistical analyses were performed using IBM SPSS Statistics for Windows, Version 20.0 (2011). For statistical analyses, n was defined as the number of replicate community populations. Independent-sample t-tests were applied to test for differences in growth rates among community types, using Bonferroni correction for multiple tests when examining the growth rates involving pure wildtype strains of each *C. elegans*, *C. briggsae* and *C. nigoni* (α = 0.025; Fig. 1). One-sample t-tests were applied to arcsine transformed data to test for differences between the initial and final frequencies of individual strains in mixed strain communities. An ANOVA was applied to test for differences in population density among community types.

## Results

To test whether harm induced by *C. nigoni* male sperm affects other species at the demographic level, we assessed populations of *C. elegans* or *C. briggsae* mixed with *C. nigoni*. By inferring species relative abundance and growth rate (*r* in Eq. 2 or *r*′ in Eq. 4) under conditions of non-limiting resources for these communities across six generations, we tested for ‘demographic RI’. Moreover, we genetically manipulated the mode of reproduction for *C. elegans* and *C. briggsae* to create three distinct treatments that differed in their likelihood of receiving male sperm (high for a feminized obligate dioecious strain, moderate for wildtype hermaphrodites, and low for vulvaless obligate selfing hermaphrodites) to explore how sensitive is the response to this gametic mechanism of interference competition to the risk of heterospecific sperm exposure.

First, we confirmed that the intrinsic population growth rate of the obligately outcrossing species *C. nigoni* is lower than the population growth rate of both *C. elegans* and *C. briggsae* when each species was grown in isolation (*C. elegans*: t = 15.7, df = 18, P ≤ 0.001; *C. briggsae*: t = 17.0, df = 17, P ≤ 0.001; Fig. 1a, d). This result affirms that *C. elegans* and *C. briggsae* should be superior resource competitors than *C. nigoni* if resources were limiting. We also saw that wildtype hermaphrodites of *C. briggsae* had a higher growth rate than *C. elegans* (t = 3.05, df = 18, P = 0.007), consistent with the experimental temperature (25 °C) being more favourable for *C. briggsae* [48]. Communities that consisted purely of *C. nigoni* showed similar growth rates in experiments involving both *C. briggsae* and *C. elegans* (ANOVA: t = 0.17, df = 17, P = 0.87; Fig. 1a, d) and reached similar densities (Additional file 1: Figure S1). That *C. nigoni* imposes component RI on *C. elegans* and *C. briggsae*, but not the reciprocal [28], motivated us to test whether reproductive interference might partly or completely offset the direct competitive disadvantage of *C. nigoni*.

**Fig. 2:**
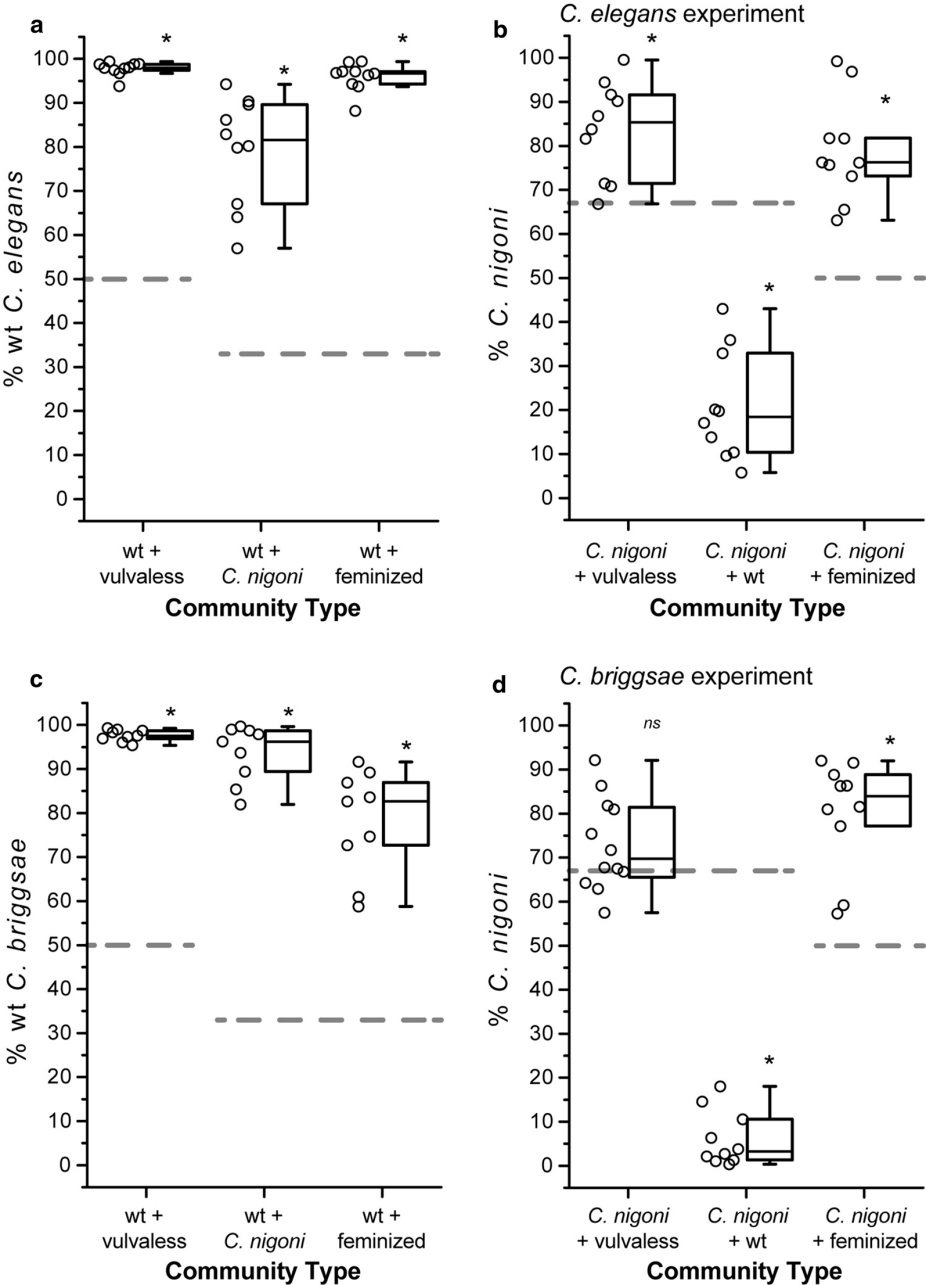
Relative frequencies of species and strain phenotypes changed in communities over time. Frequencies of species and strain phenotypes in mixed communities after six generations for experimental blocks with *C. elegans* (**a**, **b**) and *C. briggsae* (**c**, **d**). Wildtype (wt) strains of *C. elegans* increased in frequency regardless of which other strain phenotype or species they were paired with (**a** community mix with vulvaless *t*(9) = 42.07, P ≤ 0.001; *C. nigoni t*(9) = 10.19, P ≤ 0.001; feminized *t*(9) = 30.57, P ≤ 0.001), as was also true for *C. briggsae* (**c** community mix with vulvaless *t*(9) = 37.71, P ≤ 0.001; *C. nigoni t*(9) = 18.02, P ≤ 0.001; feminized *t*(9) = 10.82, P ≤ 0.001). By contrast, the feminized phenotype of both *C. elegans* and *C. briggsae* declined in frequency when grown with *C. nigoni* (**b** *C. elegans t*(9) = − 5.82, P ≤ 0.001; **d** *C. briggsae t*(9) = − 7.12, P ≤ 0.001). We observed lower frequencies of vulvaless *C. elegans* hermaphrodites when mixed with *C. nigoni*, as well (**b** *t*(9) = − 4.23, P = 0.002), although the relative frequency of vulvaless *C. briggsae* was not significantly reduced over time when reared with *C. nigoni* (**d** *t*(11) = − 2.11, P = 0.059). Asterisks (*) indicate significant differences (α = 0.05) between starting phenotype frequencies (dashed lines) and ending phenotype frequencies (ns indicates P > 0.05) from one-sample t-tests after arcsin transform. The values shown for the “wt + *C. nigoni*” community type in **a** and **b** correspond to the same underlying data and test of significance; values for “wt + *C. nigoni*” in **c** and **d** correspond to the same data and test of significance

We next sought to determine whether interspecies interactions in mixed assemblages of species would change the species-specific growth rate estimates when food was not limited, thus revealing competitive interference. When mixed with *C. nigoni*, the *C. elegans* strain with the wildtype hermaphrodite reproductive mode showed significantly slower population growth than when reared in isolation (F_3, 36_ = 47.63, P ≤ 0.001; t = 4.22, df = 18, P ≤ 0.001; Fig. 1a, d). This result is consistent with *C. elegans* suffering from demographic reproductive interference by *C. nigoni* males. Despite the reduced population growth rate of *C. elegans* wildtype hermaphrodites mixed with *C. nigoni*, they still increased in relative frequency over time, indicating that the negative interspecies interaction was not sufficient to fully offset the intrinsic growth advantage of *C. elegans* over *C. nigoni* given their relative starting frequencies (Fig. 2a; Additional file 1: Figure S2A). We also observed significantly faster population growth of *C. nigoni* in the mixed-species community with *C. elegans* than when grown alone (t =  − 3.86, df = 18, P ≤ 0.001; Fig. 1a), suggesting the possibility of facilitation by an unknown mechanism. Mixed species communities of *C. nigoni* with *C. briggsae* that had the wildtype hermaphrodite reproductive mode, however, exhibited no difference in population growth rate for either species compared to when they were reared in isolation (*C. briggsae* t =  − 0.03, df = 12.25, P = 0.98; *C. nigoni* t = 0.03, df = 11.25, P = 0.98; Fig. 1d). This result implies that wildtype *C. briggsae* did not suffer from demographic reproductive interference by *C. nigoni* males, despite strong ‘component RI’ effects [28].

We hypothesized that if mating is obligatory for reproduction, then a species would be more likely to experience interspecies errors in mating and be exposed to the potential influence of harmful gametic effects from *C. nigoni*. Consequently, we constructed species assemblages of *C. nigoni* mixed with feminized populations of *C. elegans* or *C. briggsae* that had been genetically manipulated to be composed of females and males that reproduce via obligate outcrossing instead of by self-fertilizing hermaphrodites. Estimates of the population growth rate for these feminized *C. briggsae* populations were significantly slower when grown with *C. nigoni* than when feminized *C. briggsae* were grown with wildtype hermaphrodite *C. briggsae* (t =  − 10.91, df = 18, P ≤ 0.001; Fig. 1f). This observation is consistent with demographic RI by *C. nigoni* males reducing *C. briggsae* growth rates under conditions where *C. briggsae* must mate in order to reproduce. However, feminized *C. elegans* only showed a non-significant trend of reduced population growth rates by the presence of *C. nigoni* (t =  − 1.76, df = 18, P = 0.095; Fig. 1c), suggesting a weaker magnitude of effect on *C. elegans* than *C. briggsae*.

Finally, we hypothesized that a species incapable of being inseminated would be less susceptible to demographic RI by harmful effects of gametes from another species. Therefore, we constructed species assemblages with *C. nigoni* mixed with populations of *C. elegans* and *C. briggsae* that were genetically manipulated so that hermaphrodites lacked a vulva and so could not be inseminated; vulvaless animals reproduce obligately by selfing. Indeed, we observed no significant difference in our estimates of population growth rate for vulvaless *C. elegans* when mixed with *C. nigoni* versus when grown with wildtype hermaphrodite *C. elegans* (t =  − 1.59, df = 18, P = 0.13; Fig. 1b). Estimates of population growth rate for vulvaless *C. briggsae*, by contrast, were significantly lower in the presence of *C. nigoni* than when reared with wildtype *C. briggsae* (t =  − 2.22, df = 20, P = 0.038; Fig. 1e). This latter observation suggests that some form of competitive interference mediated by a mechanism other than *C. nigoni* sperm influenced the fitness of vulvaless *C. briggsae* individuals. The proportion of vulvaless (and feminized) *C. elegans* strains declined from initial frequencies when in presence of either conspecifics or dioecious *C. nigoni* (Fig. 2a, b; Additional file 1: Figure S2A), with the disadvantage of dioecious *C. elegans* relative to hermaphrodite *C. elegans* recapitulating previous findings [49, 50]. Similarly, the proportion of feminized *C. briggsae* also declined from initial frequencies when in the presence of conspecifics or dioecious *C. nigoni* (Fig. 2b), whereas vulvaless *C. briggsae* hermaphrodites only declined significantly when mixed with wildtype conspecifics (Fig. 2b).

## Discussion

### Gametic vs. non-gametic interference competition

Our multi-species experimental communities demonstrate that the population growth rate of a given species can suffer from the presence of another species, even under conditions of non-limiting resource availability. These findings implicate some form of interference competition mediating interspecies interactions, with *Caenorhabditis* nematode biology pointing to reproductive interference as the predominant source [10, 28, 51]. We designed the communities of *Caenorhabditis* to include species combinations with inherent asymmetry in their resource competitive ability versus ability to induce harm through reproductive interactions. In particular, “component reproductive interference (RI)” by *C. nigoni* males to individual hermaphrodites of *C. elegans* and *C. briggsae* induces sterility and premature death, mediated by ectopic sperm cell migration throughout the gonad and body cavity upon interspecies matings, capable of reducing lifetime fitness by ~ 85% [28]. Consequently, we conclude that this mechanism of component RI ramifies to the population level, with “demographic reproductive interference” best explaining our observations of reduced growth rates observed in *C. elegans* and *C. briggsae* when they share experimental environments with *C. nigoni*.

In one experimental treatment, however, we detected evidence of interference competition that cannot be explained by gamete-mediated RI: populations of *C. briggsae* incapable of mating suffered reduced growth rates in the presence of *C. nigoni*. This result contrasts with our initial prediction that reduced risk of invasive heterospecific sperm would eliminate the potential for reproductive interference. We hypothesize that excessive but futile male mating attempts by *C. nigoni* on the vulvaless *C. briggsae* hermaphrodites reduced their fitness by direct physical damage or by interfering with foraging and feeding. Male nematodes continuously trace the body of a potential mating partner in an attempt to locate the vulva for spicule insertion and insemination (‘circling behavior’) [32, 52], leading to cuticular damage and, potentially, attempted erroneous copulation with the excretory pore [27, 31, 53, 54]. Moreover, as eggs hatch inside a vulvaless animal, her locomotion becomes compromised which could hamper normal hermaphrodite ability to evade male mating attempts [27, 45, 55], thus exacerbating the potential negative direct effects of persistent male courtship. Continual hermaphrodite escape behavior in the face of persistent male courtship also would limit foraging, thus also compromising resource uptake. Secretions by *Caenorhabditis* males also have been shown to alter the physiology and reduce the longevity of conspecific hermaphrodites, even in the absence of any physical interactions [30, 56]. Consequently, such non-gametic forms of pre-copulatory reproductive interference could explain the reduced growth rate of vulvaless *C. briggsae* in the presence of *C. nigoni* and might also contribute a source of interference competition in other circumstances as well.

We observed distinct responses to the presence of *C. nigoni* for *C. elegans* and *C. briggsae* when subjected to analogous mating system treatments. Given that selfing hermaphroditism evolved independently in each of these species, the distinct effects may reflect independently evolved tactics in response to male mating attempts and sperm activity [57–60]. *C. briggsae* is more closely-related to *C. nigoni* than is *C. elegans* [60]. Moreover, the geographic range of *C. briggsae* overlaps that of tropical *C. nigoni*, unlike for the temperate range of *C. elegans*; encounters with heterospecific *Caenorhabditis* might be more common in nature for tropical species in general because *Caenorhabditis* are more speciose in the tropics [33, 60, 61]. Consequently, we might expect stronger reproductive character displacement to have evolved in *C. briggsae* in the form of mate discrimination or other pre-mating isolation barriers [7]. If present, then stronger pre-mating barriers to interspecies mating could explain the lack of evidence for demographic RI for wildtype *C. briggsae* and its presence for the treatment with obligatory outbreeding *C. briggsae* (and the converse pattern for *C. elegans*). The pre-mating barriers might be insufficient to offset the exposure to greater risk of mating with heterospecifics for the obligately outbreeding *C. briggsae* treatment, making the populations experience a greater influence of the strong component RI once interspecies mating took place. While matings can occur readily between many *Caenorhabditis* species, few quantitative data have tested the degree to which pre-mating barriers might modulate inter-species mating risk [28, 62, 63].

Future work that formally tests for responses of species pairs with allopatric versus sympatric distributions would prove valuable in testing for the generality of reproductive interference to mediate reproductive character displacement and pre-mating reproductive isolation. As yet, however, few studies have demonstrated much evidence of mate choice among *Caenorhabditis* species [28, 62, 63]. Similarly, the phylogeographically-partitioned genetic variation in *C. briggsae* could prove fruitful in describing the genetic basis to any within-species genetic variation in pre-mating barriers or in susceptibility to sperm invasion [64, 65]. It also will be important to determine whether sperm as a mechanism of interspecies harm represents only an incidental weapon, or whether gametic reproductive interference has been co-opted more actively as an adaptive trait mediating competitive encounters between species (cf. allelopathy between plant species [66]).

### Frequency dependence in RI

Our experiments initialized communities with similar relative abundances of the species. However, the potential for demographic RI to control the dynamics of community composition is stronger for more skewed relative abundances, due to its frequency dependent effects [13, 15, 67]. This feature essentially describes a ‘priority effect’, a common feature of ecological communities whereby the attributes of established individuals determine the ability for later arrivals to invade or coexist [e.g. 15, 68–70]. As a result, it should be easier to detect demographic RI when the starting frequencies of species are highly skewed because fitness loss from RI will be amplified when the rarer species experiences proportionally more harmful heterospecific encounters [70–72]. Consequently, our experimental design is conservative with respect to being able to detect demographic RI, which likely explains why we observed demographic RI for only some experimental treatments. Future work that disentangles the potential influences of frequency dependent effects, pre-mating barriers, and species differences will be valuable in fully deciphering the importance of demographic RI in mediating species coexistence.

Importantly, previous findings of component RI in *Caenorhabditis* generally used highly male skewed sex ratios (1 hermaphrodite: 6 heterospecific males) and only gave *C. nigoni* males the option of mating with heterospecific females, which could inflate the incidence of interspecies mating relative to when a choice of conspecific versus heterospecific mates are available [28]. Even those conditions favorable to interspecies mating yielded < 50% interspecies mating success after 18–24 h (unpublished observations), reflecting the facts that *C. nigoni* females are more likely than *C. briggsae* and *C. elegans* hermaphrodites to mate with *C. nigoni* males [28] and that hermaphrodites are behaviourally uncooperative, even with conspecific males [27]. Despite these factors that should limit our ability to detect demographic reproductive interference, we nevertheless observed repeated cases of reduced population growth rates of *C. elegans* and *C. briggsae* when they co-occurred with *C. nigoni*. Future studies with *Caenorhabditis* that build on our proof-of-principle demonstration of demographic RI should further characterize it by testing additional species communities and by varying the relative abundance in the composition of founding communities [70].

Although our experimental design largely precludes the possibility of evolution, we anticipate that sperm-mediated reproductive interference could drive trait evolution within species. Future studies with *Caenorhabditis* can address directly this issue [73]. Specifically, we expect that the strength of reproductive interference, and correspondingly the strength of selection, would be greater when one species is rarer because of more intense exposure to harmful interactions, provided that the cost is not so great as to drive it locally extinct. As for other mechanisms of reproductive character displacement [24], we should expect more frequent encounters between species that exact sperm-mediated harm during sexual interactions to lead to the faster evolution of stronger pre-mating reproductive isolation [74]. It remains unclear how sensitive to encounter frequency would be the coevolutionary dynamics between species in causing and evading harm from reproductive interference. While sympatry-allopatry comparisons of reproductive traits provide a classic way to evaluate the consequences of interspecies interactions, quantitative variation in the likelihood of interspecies encounters present a greater challenge for prediction [74]. Theory shows that even small amounts of harmful reproductive interactions between species can confer large effects on trait evolution and coexistence relative to direct resource competition [74]. *Caenorhabditis* provides an experimentally tractable system for future tests of additional features of coevolutionary and coexistence theory. These basic principles generalize to systems beyond nematodes and their particular mechanism of reproductive interference [7–9], permitting experimental interrogation of theory about ecoevolutionary processes.

## Conclusion

Our laboratory experiments with *Caenorhabditis* nematodes demonstrate how interspecies reproductive interference between individuals can ramify to influence population demography, with the potential to alter species community structure and trait evolution. The mechanisms of reproductive interference involve negative effects of ectopic sperm migration throughout the female or hermaphrodite body [28], with a potential additional contribution of physical harm and food deprivation from continuous escape behavior induced by heterospecific mating attempts [27, 31]. It remains unknown, however, how frequently species of *Caenorhabditis* interact with one another in nature. The intensifying study of the natural context of *Caenorhabditis* nematodes [33, 37, 48, 61, 75, 76], along with naturalistic experimental microcosms [73, 77], make them promising for relating species coexistence theory, mechanisms of reproductive interference, antagonistic coevolution and reproductive character displacement, and community dynamics in nature. With rapidly changing and human-impacted environments around the world, organisms that have historically never interacted with one another are being brought together at an unprecedented pace [78], including invasive species with small initial populations relative to native population sizes [79]. Therefore, full consideration of reproductive interference as a mode of interspecies interactions is crucial for elucidating a general understanding of biodiversity and ecological functioning, with *Caenorhabditis* offering a powerful system for manipulative study.

## Additional files

**Additional file 1: Figure S1.** Density of communities at the end of the experiment. **Figure S2.** Change in the relative frequency of phenotypes or species in different community types.

**Additional file 2.** Raw data for *Caenorhabditis* reproductive interference experiments.

## Abbreviations

RI: reproductive interference
GFP: green fluorescent protein
