# Supplementary material for: Demographic consequences of reproductive interference in multi-species communities
Source: BMC Ecol. 2018 Nov 6;18:46. doi: 10.1186/s12898-018-0201-0 (PMC6219154; doi:10.1186/s12898-018-0201-0)
Supplement: Supplementary file 1 — Additional file 1: Figure S1. Density of communities at the end of the experiment. Figure S2. Change in the relative frequency of phenotypes or species in different community types. [file 12898_2018_201_MOESM1_ESM.pdf]

Supplementary Figures

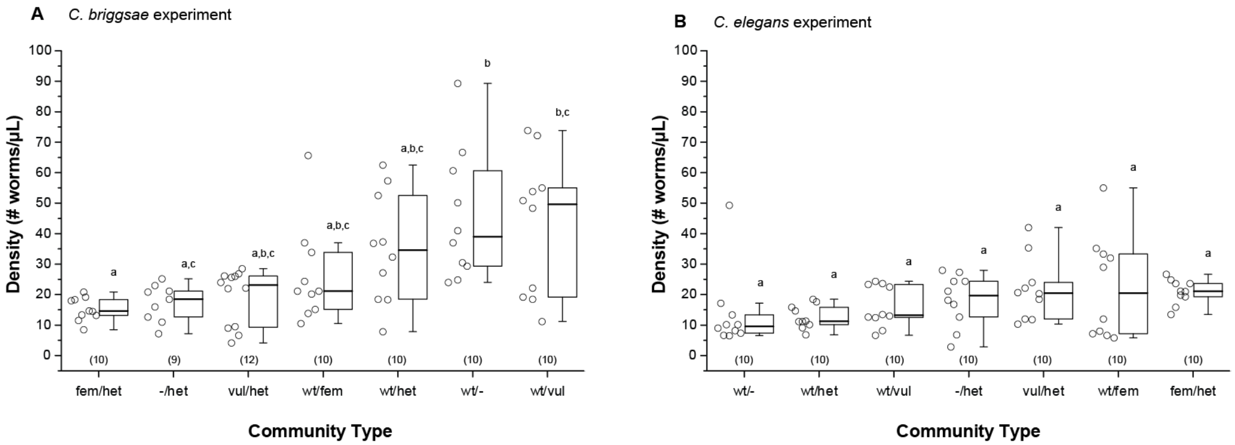

Figure S1. Density of communities at the end of the experiment.

Final estimated density (worms/µL from 2 µL aliquots) differed among communities in **(A)** the *Caenorhabditis briggsae* experiment (Kruskal-Wallis: χ^2=26.9, df=6, p≤0.001), and in **(B)** the *C. elegans* experiment (χ^2=12.8, df=6, p=0.046). Overall, in the *C. briggsae* experiment communities had higher densities when compared to communities in the *C. elegans* experiment (Kruskal-Wallis: χ^2=13.9, df=1, p≤0.001). Boxplot whiskers indicate 1.5*(interquartile range), open circles represent the estimated density of each population from the different community types, and the number of replicates in each community type are in parentheses. Lower cases letters indicate differences following Bonferroni correction for multiple post-hoc tests (α = 0.008). Abbreviations for phenotypes: (–) absence of a second phenotype, (*wt*) wildtype hermaphrodites, (*het*) heterospecific (*C. nigoni*), (*vul*) vulvaless hermaphrodites (unable to receive male sperm), (*fem*) feminized hermaphrodites (require male sperm to reproduce).

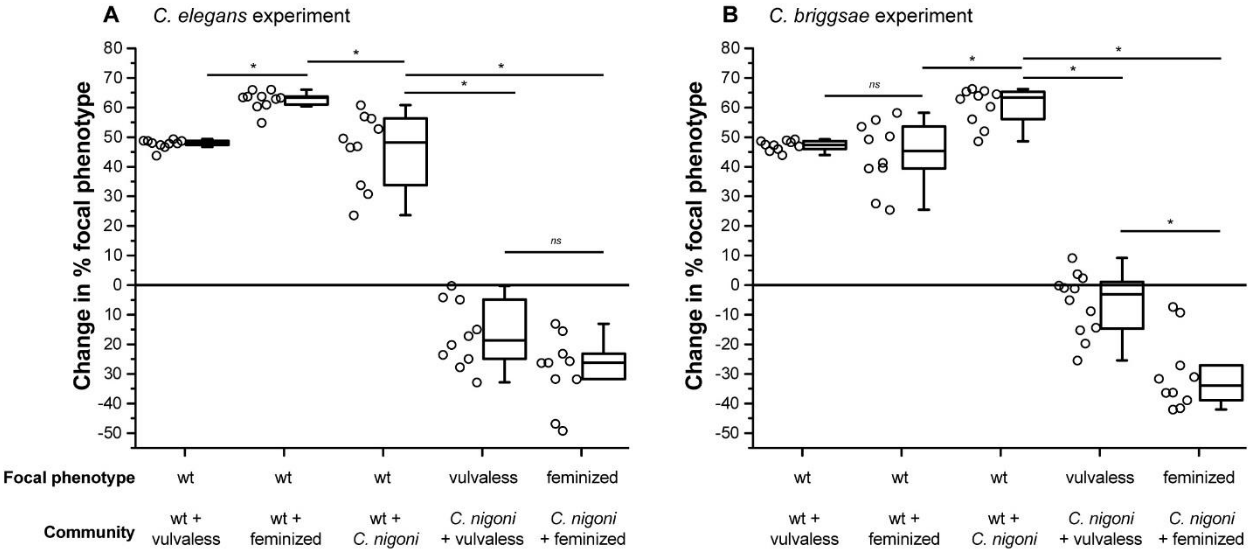

Figure S2. Change in the relative frequency of phenotypes or species in different community types. Frequencies of species and strain phenotypes in mixed communities after six generations for experimental blocks with **(A)** *C. elegans* and **(B)** *C. briggsae* (see Figure 2 in main text for observed frequencies and statistical details). Boxplot whiskers indicate 1.5*(interquartile range), open circles represent the estimated change in frequency (%) of a phenotype between the start and end of the study of each population from the different community types.
